# Clinical characteristics, activity levels and mental health problems in children with long coronavirus disease: a survey of 510 children

**DOI:** 10.2217/fmb-2021-0285

**Published:** 2022-04-01

**Authors:** Danilo Buonsenso, Ferran Espuny Pujol, Daniel Munblit, Davide Pata, Sammie McFarland, Frances K Simpson

**Affiliations:** 1 Department of Woman and Child Health and Public Health, Fondazione Policlinico Universitario A Gemelli Istituto di Ricovero e Cura a Carattere Scientifico, Rome, Italy; 2 Dipartimento di Scienze Biotecnologiche di Base, Cliniche Intensivologiche e Perioperatorie, Università Cattolica del Sacro Cuore, Rome, Italy; 3 Global Health Research Institute, Istituto di Igiene, Università Cattolica del Sacro Cuore, Roma, Italy; 4 Clinical Operational Research Unit, University College London, London, UK; 5 Department of Paediatrics and Paediatric Infectious Diseases, Institute of Child's Health, Sechenov First Moscow State Medical University, Moscow, Russia; 6 Inflammation, Repair and Development Section, National Heart and Lung Institute, Faculty of Medicine, Imperial College London, London, UK; 7 Long COVID Kids, UK; 8 Coventry University Group, Coventry, UK

**Keywords:** children, long COVID, neuropsychiatric symptoms, prolonged symptoms, survey

## Abstract

**Background:**

Whether long coronavirus disease pertains to children as well is not yet clear.

**Methods:**

The authors performed a survey in children suffering from persistent symptoms since initial infection. A total of 510 children infected between January 2020 and January 2021 were included.

**Results:**

Symptoms such as fatigue, headache, muscle and joint pain, rashes and heart palpitations and issues such as lack of concentration and short-term memory problems were particularly frequent and confirm previous observations, suggesting that they may characterize this condition.

**Conclusion:**

A better comprehension of long coronavirus disease is urgently needed.

One year after the first description of SARS-CoV-2 in China, several milestones were achieved in the understanding of the epidemiological and physiopathological bases of COVID-19 [1] and its treatment [2], and a number of effective vaccines have been developed and marketed [3]. However, unexplained issues still remain. Among these has been the recognition that a relevant percentage of patients with COVID-19 experience persistent symptoms after resolution of the acute phase of the disease, which is often characterized by the ‘classic triad’ of cough, fever and anosmia. Although patients relatively quickly began highlighting their persistent symptoms and change in quality of life, Carfì *et al.* first documented this in an international peer-reviewed journal [4]. The Italian researchers found that in patients who had recovered from COVID-19, 87.4% reported persistence of at least one symptom, particularly fatigue and dyspnea. Later, several other articles confirmed these data in adults, and a recent large cohort of 1733 patients from Wuhan found persistent symptoms in 76% of patients 6 months after initial diagnosis [5]. Since this condition is still not completely understood, a definite official name was not initially recognized. Nevertheless, patient organizations started complex discussions and movements on social media with various kinds of evidence and advocacy to demonstrate a longer, more complex course of illness than laid out in initial reports from Wuhan and eventually coined the term ‘long COVID’ [6], which was later also recognized by WHO [7].

Researchers have only recently begun to study why people develop these symptoms. According to WHO, several explanations can be considered [7]: persistence of the virus in some parts of the body that are sheltered from the immune system, such as the brain; direct damage to organs, such as the heart and lungs as well as the pancreas, causing some new cases of diabetes; and blood clotting, which can cause heart attacks and strokes. Other mechanisms have also been suggested, including a dysregulated immune response to the infection, which can include mast cell activation [8], autoimmunity or changes in the autonomic nervous system [9]. However, there is a huge variety in both the pattern of symptoms and their severity, with sex and, possibly, age differences.

Although at the beginning children were considered relatively spared by the pandemic, around December 2020 parent movements began highlighting that some children had prolonged symptom after acute COVID-19. Parents developed social media movements aiming to highlight that children were also suffering from long COVID. Long COVID Kids UK [20] began initially with the launch of an online message on YouTube [21] on 31 October 2020, and with Facebook and Twitter channels. Since its start, 197,000 people have been reached on Facebook and 1.8 million interactions have occurred on Twitter. Thus far, the group includes over 1800 children from 1332 families (Supplementary Figures).

Subsequently, a case series from Sweden described a group of five children with long COVID [10]. A larger study from Italy confirmed that about one of three children with acute COVID-19 experienced persistent symptoms months after initial diagnosis [11]. Considering the importance of patient-driven data in this pandemic, and for the understanding of long COVID in particular, and aiming to provide more insights into the burden of long COVID in children, one of the largest parent movements (Long COVID Kids UK) performed an online follow-up survey of a large cohort of children who experienced COVID-19 and had persistent symptoms.

## Methods

### Long COVID Kids Rapid Survey 2

To assess the presence of persistent symptoms in children with previous COVID-19, the parents nonprofit association Long COVID Kids developed an online platform where parents from all over the world can access and anonymously report their child's experience. The ‘Long COVID Kids Rapid Survey 2’ was designed as a follow-up to a pilot survey (that established quantity and type of symptoms) as a means to establish clusters of symptoms rather than the full breadth of symptoms as well as the effects on the mental and physical health of the child as a result of long COVID. Certain symptoms were deliberately excluded, as they were not considered relevant to the clusters under consideration. Links to the survey site on Jotform were disseminated on the closed Facebook group ‘LongCOVIDKids’. Parents' consent was required before answering questions regarding their children with persistent COVID-19 symptoms.

In the ‘Long COVID Kids Rapid Survey 2’, participants were asked to self-declare the following main information on their children: how COVID-19 was confirmed and details at infection, including need for hospitalization, age, sex and ethnicity; month of initial infection; course of symptoms, if any, from initial infection; activity before and after infection; mental health status and comorbidities before COVID-19; displayed symptoms since COVID-19; behavior/activity/habit changes after COVID-19; need for medical care after COVID-19; parents' perspective of need for medical care for their children and type of care; and parents' perspective on child's need of support to be readmitted to school after COVID-19 [22].

For the purpose of this study, the authors used data from the ‘Long COVID Kids Rapid Survey 2’ collected between 13 February 2021 and 6 March 2021. Only those children with symptoms lasting longer than 4 weeks were included. The survey was independently and autonomously developed by parents of children with COVID-19 and made open access and anonymous on the website. Parents were requested to give their consent to participate anonymously. Given that the survey was a parent-led initiative and parents were active participants in both research and authorship, ethics committee approval was not necessary.

### Data analysis

The confirmation status of COVID-19 infection was confirmed using the question, ‘Has your child had confirmed or suspected COVID-19 infection?’ The possible answers to that question were the following: ‘a clinical diagnosis was made by a medical professional in the absence of a confirmatory test (clinical diagnosis)’; ‘positive (antibody) lateral flow test (lateral flow)’; ‘positive polymerase chain reaction (PCR) swab’ and ‘unconfirmed by a test or medical professional, but we think we had it’. The authors will initially report the counts for the original possible answers and will use a simplified version in the tables by merging ‘positive PCR swab’ and ‘lateral flow’ into ‘positive test’. Time from infection was estimated (with a 15-day uncertainty) by subtracting the 15th day of the reported month of (confirmed or suspected) infection from the date of response to the survey. Children with an estimated time from infection <1.5 months were excluded to ensure that all included children had had symptoms for longer than 4 weeks. In practice, this implied excluding all children infected in February 2021 and those infected in January 2021 and reported in February 2021.

The authors produced summary tables and graphs aimed at the description of the study sample and the symptoms and changes in long COVID children, looking further at changes in physical activity levels and mental health. The authors cross-tabulated variables by confirmation status of COVID-19 infection and by the pre-existence of comorbidities. Analyses were performed with R software.

## Results

### Study sample

**Figure 1.**
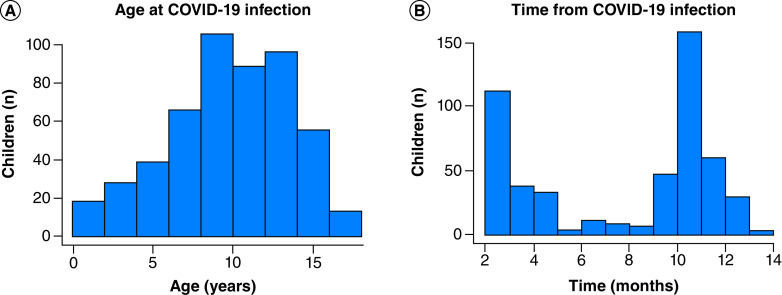
Age of respondents at COVID-19 infection (A) and time from infection at date of response to the ‘Long COVID Kids Rapid Survey 2’ questionnaire (B).

Data on 510 children who had had COVID-19 for more than 4 weeks were reported by their parents. A total of 351 (68.8%) children lived in the UK and 94 (18.4%) lived in the USA. The children contracted COVID-19 between January 2020 and January 2021 at a mean age of 10.3 years (standard deviation: 3.8) and IQR age of 10 years (8–13), with an age range of 1–18 years (Figure 1). A total of 287 (56.3%) children were female. For 297 (58.2%) children, COVID-19 was confirmed by a positive PCR test (n = 141), positive (antibody) lateral flow test (n = 4) or clinical diagnosis (n = 156). For 209 (41%) children, COVID-19 was suspected but had not been confirmed by a test or medical professional. Most of these children were from the UK and were infected around March 2020, at a time when access to tests, particularly for nonsevere cases, was difficult in most countries (Table 1).

**Table 1.** Confirmation status of COVID-19 infection by country of residence and time from infection.

Confirmation status	Country of residence			Time from infection		
	UK (N = 351)	USA (N = 94)	Other (N = 65)	1–2 months (N = 82)	3–6 months (N = 108)	7+ months (N = 320)
Clinical diagnosis, n (%)	100 (28.5)	39 (41.5)	17 (26.2)	13 (15.9)	21 (19.4)	122 (38.1)
Positive test, n (%)	83 (23.6)	37 (39.4)	25 (38.5)	64 (78)	60 (55.6)	21 (6.6)
Unconfirmed but suspected, n (%)	168 (47.9)	18 (19.1)	23 (35.4)	5 (6.1)	27 (25)	177 (55.3)

At their initial COVID-19 infection, only 22 (4.3%) children were hospitalized; 62 (12.2%) were asymptomatic, 378 (74.1%) were managed at home and 48 (9.4%) went to hospital but were not admitted. A total of 223 (43.7%) children had no pre-existing conditions, and the most frequent comorbidities were allergies (15.9%), asthma (14.5%), eczema (12.4%) and anxiety (7.1%). Detailed information on the children's pre-COVID conditions is available in Supplementary Table 1. A total of 411 (80.6%) children had no pre-COVID mental health concern or diagnosis.

### Persistence of symptoms in children since COVID-19

Overall, participating children had had persistent COVID-19 for a mean of 8.2 months (standard deviation: 3.9) (Figure 1). Details of reported symptoms are described in Table 2. The most frequent symptoms were tiredness and weakness (444 children; 87.1% of sample), fatigue (410 children; 80.4% of sample), headache (401 children; 78.6% of sample), stomach pain or cramps (387 children; 75.9% of sample), muscle aches and pains (349 children; 68.4% of sample), muscle and joint pain (309 children; 60.6% of sample), postexertional malaise (274 children; 53.7% of sample), rash (267 children; 52.4% of sample), unexplained irritability (262 children; 51.4% of sample) and dizziness (245 children; 48% of sample). A total of 484 (94.9%) children had at least four symptoms. Supplementary Table 2 reports symptoms according to disease diagnosis and pre-existing conditions.

**Table 2.** Symptoms present since COVID-19 infection by pre-existing conditions, sex, age and time from infection.

Symptom	All (N = 510)	Pre-existing conditions (N = 287)	No pre-existing conditions (N = 223)	Female (N = 287)	Male (N = 222)	Age <10 years (N = 196)	Age ≥10 years (N = 314)	1–2 months (N = 82)	3–6 months (N = 108)	7+ months (N = 320)
Cardiorespiratory, n (%)										
– Heart palpitations	205 (40.2)	112 (39)	93 (41.7)	121 (42.2)	83 (37.4)	71 (36.2)	134 (42.7)	29 (35.4)	45 (41.7)	131 (40.9)
– Coughing	151 (29.6)	81 (28.2)	70 (31.4)	79 (27.5)	71 (32)	66 (33.7)	85 (27.1)	25 (30.5)	30 (27.8)	96 (30)
– Throat clearing	107 (21)	59 (20.6)	48 (21.5)	59 (20.6)	47 (21.2)	55 (28.1)	52 (16.6)	21 (25.6)	16 (14.8)	70 (21.9)
Dermatological, n (%)										
– Rash	267 (52.4)	149 (51.9)	118 (52.9)	143 (49.8)	123 (55.4)	118 (60.2)	149 (47.5)	35 (42.7)	54 (50)	178 (55.6)
– Red and cracked lips	201 (39.4)	112 (39)	89 (39.9)	124 (43.2)	77 (34.7)	89 (45.4)	112 (35.7)	33 (40.2)	37 (34.3)	131 (40.9)
– Peeling skin on hands and feet	143 (28)	83 (28.9)	60 (26.9)	78 (27.2)	65 (29.3)	63 (32.1)	80 (25.5)	20 (24.4)	26 (24.1)	97 (30.3)
– Swollen hands and feet	107 (21)	56 (19.5)	51 (22.9)	62 (21.6)	45 (20.3)	42 (21.4)	65 (20.7)	10 (12.2)	14 (13)	83 (25.9)
– Ulcers	79 (15.5)	43 (15)	36 (16.1)	51 (17.8)	28 (12.6)	47 (24)	32 (10.2)	10 (12.2)	11 (10.2)	58 (18.1)
Gastrointestinal, n (%)										
– Stomach pain or cramps	387 (75.9)	218 (76)	169 (75.8)	225 (78.4)	161 (72.5)	157 (80.1)	230 (73.2)	58 (70.7)	78 (72.2)	251 (78.4)
– Nausea	233 (45.7)	134 (46.7)	99 (44.4)	135 (47)	97 (43.7)	78 (39.8)	155 (49.4)	35 (42.7)	52 (48.1)	146 (45.6)
– Diarrhea and vomiting	216 (42.4)	128 (44.6)	88 (39.5)	115 (40.1)	100 (45)	92 (46.9)	124 (39.5)	34 (41.5)	44 (40.7)	138 (43.1)
HEENT, n (%)										
– Red eyes	206 (40.4)	113 (39.4)	93 (41.7)	102 (35.5)	104 (46.8)	87 (44.4)	119 (37.9)	30 (36.6)	41 (38)	135 (42.2)
– Sore throat	230 (45.1)	132 (46)	98 (43.9)	131 (45.6)	99 (44.6)	87 (44.4)	143 (45.5)	36 (43.9)	43 (39.8)	151 (47.2)
– Swollen neck glands	128 (25.1)	74 (25.8)	54 (24.2)	81 (28.2)	47 (21.2)	46 (23.5)	82 (26.1)	19 (23.2)	21 (19.4)	88 (27.5)
Musculoskeletal, n (%)										
– Muscle aches and pains	349 (68.4)	204 (71.1)	145 (65)	201 (70)	147 (66.2)	111 (56.6)	238 (75.8)	60 (73.2)	70 (64.8)	219 (68.4)
– Muscle and joint pain	309 (60.6)	179 (62.4)	130 (58.3)	180 (62.7)	129 (58.1)	102 (52)	207 (65.9)	51 (62.2)	69 (63.9)	189 (59.1)
Neurological, n (%)										
– Headache	401 (78.6)	239 (83.3)	162 (72.6)	231 (80.5)	169 (76.1)	138 (70.4)	263 (83.8)	63 (76.8)	86 (79.6)	252 (78.8)
– Unexplained irritability	262 (51.4)	149 (51.9)	113 (50.7)	150 (52.3)	112 (50.5)	117 (59.7)	145 (46.2)	41 (50)	56 (51.9)	165 (51.6)
– Dizziness	245 (48)	148 (51.6)	97 (43.5)	150 (52.3)	94 (42.3)	60 (30.6)	185 (58.9)	39 (47.6)	55 (50.9)	151 (47.2)
– Twitches	55 (10.8)	34 (11.8)	21 (9.4)	35 (12.2)	20 (9)	16 (8.2)	39 (12.4)	9 (11)	10 (9.3)	36 (11.2)
– Word repetition	52 (10.2)	25 (8.7)	27 (12.1)	29 (10.1)	22 (9.9)	21 (10.7)	31 (9.9)	7 (8.5)	14 (13)	31 (9.7)
– Tics	47 (9.2)	25 (8.7)	22 (9.9)	26 (9.1)	21 (9.5)	18 (9.2)	29 (9.2)	5 (6.1)	8 (7.4)	34 (10.6)
– Stuttering	40 (7.8)	17 (5.9)	23 (10.3)	23 (8)	17 (7.7)	15 (7.7)	25 (8)	4 (4.9)	7 (6.5)	29 (9.1)
– Swearing	26 (5.1)	15 (5.2)	11 (4.9)	9 (3.1)	17 (7.7)	10 (5.1)	16 (5.1)	2 (2.4)	5 (4.6)	19 (5.9)
– Growling	24 (4.7)	12 (4.2)	12 (5.4)	11 (3.8)	12 (5.4)	15 (7.7)	9 (2.9)	5 (6.1)	4 (3.7)	15 (4.7)
General, n (%)										
– Tiredness and weakness	444 (87.1)	249 (86.8)	195 (87.4)	248 (86.4)	195 (87.8)	168 (85.7)	276 (87.9)	70 (85.4)	98 (90.7)	276 (86.2)
– Fatigue	410 (80.4)	235 (81.9)	175 (78.5)	236 (82.2)	173 (77.9)	142 (72.4)	268 (85.4)	64 (78)	93 (86.1)	253 (79.1)
– Postexertional malaise	274 (53.7)	161 (56.1)	113 (50.7)	158 (55.1)	116 (52.3)	95 (48.5)	179 (57)	44 (53.7)	57 (52.8)	173 (54.1)
– Fever	151 (29.6)	81 (28.2)	70 (31.4)	94 (32.8)	56 (25.2)	68 (34.7)	83 (26.4)	22 (26.8)	29 (26.9)	100 (31.2)
– Flu-like symptoms	121 (23.7)	69 (24)	52 (23.3)	78 (27.2)	42 (18.9)	47 (24)	74 (23.6)	20 (24.4)	20 (18.5)	81 (25.3)
Other, n (%)										
– Sepsis	7 (1.4)	5 (1.7)	2 (0.9)	4 (1.4)	3 (1.4)	3 (1.5)	4 (1.3)	0 (0)	1 (0.9)	6 (1.9)
– Appendicitis	7 (1.4)	6 (2.1)	1 (0.4)	5 (1.7)	2 (0.9)	4 (2)	3 (1)	0 (0)	0 (0)	7 (2.2)
– Peritonitis	1 (0.2)	0 (0)	1 (0.4)	0 (0)	1 (0.5)	0 (0)	1 (0.3)	0 (0)	0 (0)	1 (0.3)

Results were not significantly affected by age, comorbidities or sex (p > 0.05).

HEENT: Head, eyes, ears, nose and throat.

A total of 129 (25.3%) children had experienced persistent symptoms since acute disease/infection, 252 (49.4%) had periods of apparent recovery followed by return of symptoms and 97 (19%) had a prolonged period of wellness followed by symptoms. Among those who had no pre-COVID conditions, the authors found that it was slightly less common to have persistent symptoms without recovery periods (23.8 vs 26.5%) or alternating recovery/symptom episodes (48.4 vs 50.2%), although there was no difference in constant COVID-19 symptoms between those reporting pre-existing conditions (Table 3).

**Table 3.** Children's experience of COVID-19 by confirmation of infection and pre-existing conditions.

Characteristics	All (N = 510)	Clinical diagnosis (N = 156)	Positive test (N = 145)	Unconfirmed but suspected (N = 209)	Pre-existing conditions (N = 287)	No pre-existing conditions (N = 223)
Constant symptoms, n (%)	129 (25.3)	45 (28.8)	50 (34.5)	34 (16.3)	76 (26.5)	53 (23.8)
Alternating recovery/symptoms, n (%)	252 (49.4)	78 (50)	66 (45.5)	108 (51.7)	144 (50.2)	108 (48.4)
Long period of wellness followed by symptoms, n (%)	97 (19)	21 (13.5)	24 (16.6)	52 (24.9)	46 (16)	51 (22.9)
Undetermined, n (%)	32 (6.3)	12 (7.7)	5 (3.4)	15 (7.2)	21 (7.3)	11 (4.9)

Results were not significantly affected by age, comorbidities or sex (p > 0.05).

### Changes in children since COVID-19 infection

**Figure 2.**
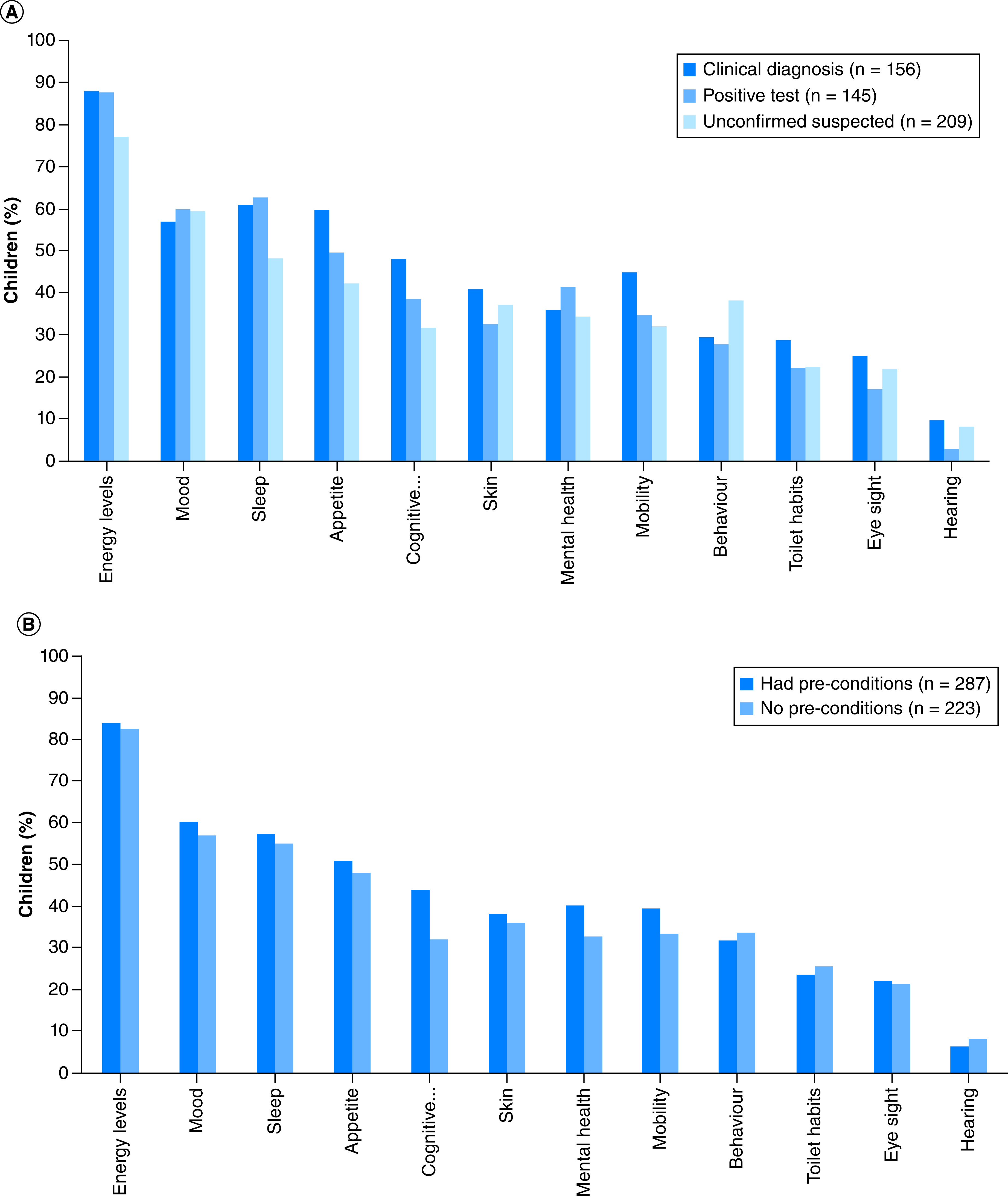
Changes reported since COVID-19 infection by confirmation of infection (A) and pre-existing conditions (B).

Long COVID children had experienced complex changes since COVID-19 infection (Figure 2 & Supplementary Table 3). The most frequently reported changes were related to energy levels (425 children; 83.3% of sample), mood (300 children; 58.8% of sample), sleep (287 children; 56.3% of sample) and appetite (253 children; 49.6% of sample). Changes in appetite were significant in children with confirmed/unconfirmed COVID-19 and occurred similarly in those with or without pre-existing conditions. Overall, all children had had at least one change, and 325 (63.7%) had had at least four changes since their COVID-19 infection. The proportion of those with at least four changes was >60% independent of whether they had had pre-COVID conditions (Supplementary Table 4).

### Changes in physical activity levels

Most children were physically active before their COVID-19 infection. During the first 6 weeks after infection, 262 (51.4%) children participated in some level of activity and 217 (42.5%) did not, and the parents of 31 (6.1%) children were unsure.

Families reported that their children's activity levels were worse than prior to infection (Supplementary Tables 5 & 6). Only 51 (10%) children had returned to previous levels of activity. A total of 108 (21.2%) children were reported to be unable to return to previous levels of activity, and 154 (30.2%) enjoyed occasional activity, but usually had an increase in symptoms after. Overall, the more physically active the children were before COVID-19, the more likely they were to return to previous activity levels, although these rates were very low, with only 17 (11.8%) children who had practiced daily sports before COVID-19 returning to previous levels.

### Changes in mental health

**Figure 3.**
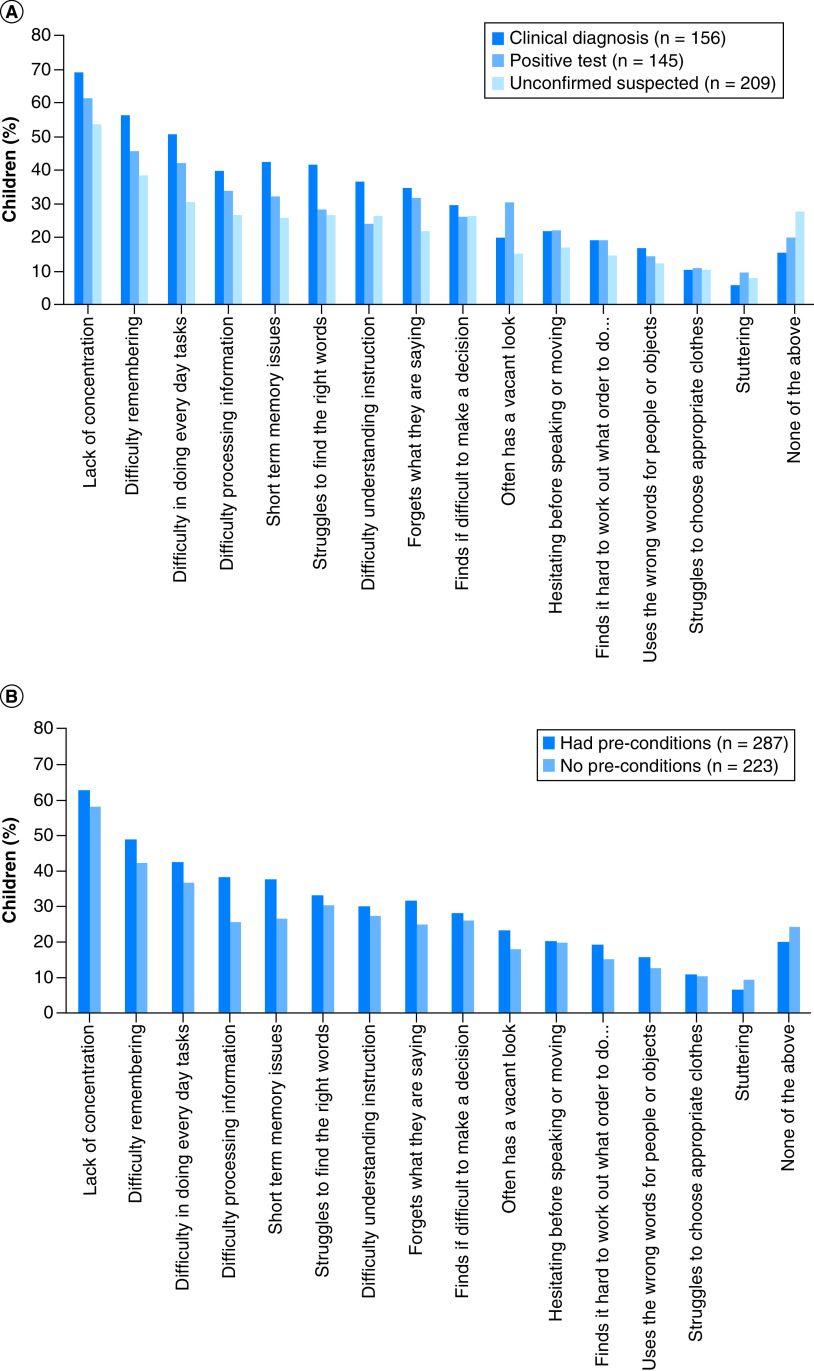
Mental health/cognitive issues since COVID-19 infection (multiple issues per child were usual). By confirmation of infection **(A)** and pre-existing conditions **(B)**.

Parents reported a significant prevalence of neuropsychiatric symptoms in children with persistent symptoms (Figure 3 & Supplementary Table 7). Specifically, several parents reported lack of concentration (309 children; 60.6% of sample), difficulty remembering information (234 children; 45.9% of sample), difficulty doing everyday tasks (204 children; 40% of sample), difficulty processing information (167 children; 32.7% of sample) and short-term memory issues (167 children; 32.7% of sample). A total of 279 (54.7%) children had had at least three mental health issues (excluding ‘none of the above’ and ‘other’), 45 (8.8%) had had two issues, 54 (10.6%) had had one issue and 132 (25.9%) had had no issues (excluding ‘none of the above’ and ‘other’). Only 64 (28.7%) of those with no pre-COVID conditions had not had any mental health/cognitive issues since their COVID-19 infection (Supplementary Table 8).

## Discussion

In this study, the authors performed a comprehensive assessment of parents' perspectives regarding the long-term physical and mental health outcomes of a large cohort of children with persistent symptoms after initial COVID-19 infection, providing details about reported symptoms, their dynamics and overall changes in children's activities and mental health. The authors found that fatigue, headache, muscle and joint pain, postexertional malaise, rashes and heart palpitations were commonly reported by parents. This cluster of symptoms was frequently reported in another pediatric long COVID study from Italy, where insomnia (18.6%), respiratory symptoms (including pain and chest tightness) (14.7%), nasal congestion (12.4%), fatigue (10.8%), muscle pain (10.1%), joint pain (6.9%) and concentration difficulties (10.1%) were the most frequently reported symptoms [11]. In the Italian study, 35 (27.1%) children had at least one symptom 120 days or more after diagnosis, and 29 of the 68 (42.6%) children assessed ≥120 days from diagnosis were still distressed by these symptoms. A similar pattern of symptoms was reported in a small case series published in Sweden by Ludvigsson [10], which reported five children with fatigue, dyspnea, heart palpitations or chest pain, headache, difficulty concentrating, muscle weakness, dizziness and sore throat months after initial diagnosis. Although some had improved after 6–8 months, none had fully returned to school. Interestingly, the Italian and Swedish patients in these studies had age patterns similar to those of the UK children in the current study. Of note, studies on long COVID in adults report a very similar pattern of symptoms as well [4,5]. The similarity of reported symptoms in the different cohorts of adult and pediatric studies suggests that these may be the defining symptoms of long COVID in general.

In this survey, the authors assessed symptom dynamics. According to descriptions from patient organizations and adult studies, this is an important aspect of long COVID, although it has not been assessed in children before. Interestingly, the authors found that 25.3% of children had experienced constant COVID-19 infection symptoms, 49.4% had had periods of apparent recovery followed by return of symptoms and 19.0% had had a prolonged period of wellness followed by return of symptoms. Importantly, a similar pattern was reported in those with and without pre-existing conditions. To date, the authors have not found any pediatric study with which to compare these data.

Since physical activity plays an important role in child health, growth, development and socialization, the authors assessed how it changed after infection with COVID-19. Overall, the authors found that most children had worse activity levels than before infection, as reflected by the fact that, at the time of the survey, 21.2% were unable to perform activity and 30.2% enjoyed occasional activity but usually had an increase in symptoms after. Although lockdown and school closure may have contributed to this change [12], recent evidence suggests that immunological dysfunction in children with long COVID may explain some of this [13]. Similarly, two recent studies have documented lung perfusion defects in children following COVID-19 infection [14,15]

The authors also assessed mental health issues in this cohort of children with previous COVID-19 infection. Over the last few months of the pandemic, healthcare professionals have raised several warnings after seeing an increase in suicide attempts and other issues related to mental health in children and adolescents [16,17]. Parents have reported a wide range of neurocognitive symptoms, including lack of concentration, difficulty processing/remembering information or understanding instructions, short-term memory issues and struggles finding the right words. Similar problems have been reported in the only pediatric study performed thus far [11] as well as in several adult studies [4,5].

This study has several limitations that must be addressed. First, it was an online survey that was shared only through an online platform and not systematically proposed to consecutively diagnosed children in specific settings and therefore demonstrates a selection bias. Since this survey was exclusively developed by parents with the original aim of informing families and professionals and not performing an original study, the survey has some intrinsic limitations, such as overlap of some categories of symptoms (i.e., tiredness and weakness vs fatigue), use of categories that conflate different constructs (i.e., tiredness usually having a different pathophysiology than weakness), no use of standardized instruments and some wording choices that may have led to lack of clarity in responses (i.e., the questionnaire speaks of ‘enjoyment’ of activities rather than the ability to perform them, which would be a more important descriptor). In addition, this survey was launched on the page of Long COVID Kids UK, which was created with the purpose of providing awareness and support to families with children with long COVID. Therefore, this represents a substantial bias since parents of children with persistent symptoms may have had more interest in participating in this survey, and this may explain the large number of children with persistent symptoms in this cohort compared with other cohorts. The absence of a systematic sampling procedure hurts the generalizability of the data, as the authors do not know what drew individuals to the website and are therefore not able to define the incidence of long COVID in children.

Another limitation is that not all children received a microbiologically confirmed diagnosis. This is mainly due to the unpreparedness of health systems [18] and difficulties in accessing testing, particularly during the first months of the pandemic, as well as the different rules and practices in use in different settings. In addition, the small number of children requiring hospitalization did not allow the authors to determine how initial severity affected long COVID in children. The lack of a control group also does not allow the authors to determine a cause–effect link between COVID-19 infection and these symptoms. However, this study represents the first effort by families of children with long COVID to highlight the persistent symptoms that affect children for months after acute infection and that can often impact on their daily routine. It is important to provide, in these cases, comprehensive multidisciplinary support to these families. As the authors have discussed elsewhere [19], it is important that doctors take long COVID seriously and let families know they are not alone during the recovery process.

## Conclusion

We described a cluster of the most frequent symptoms and their dynamics in children with long COVID in a large cohort of children. Symptoms such as fatigue, headache, muscle and joint pain, rashes and heart palpitations and mental health issues such as lack of concentration and short-term memory problems were particularly frequent and confirmed previous observations, suggesting that they may characterize this condition. A better comprehension of long COVID is urgently needed, especially considering that at the moment there are no therapeutic options for these children who, months after COVID-19, struggle to come back to a normal life.

## Future perspective

A non-negligible percentage of children develop a series of chronic symptoms after primary COVID-19 infection that characterize the condition known as long COVID. Although only 4.3% of the children in our study were hospitalized, more than half reported symptoms such as fatigue, weakness, headache and muscle and joint pain. This resulted in a reduction in their physical activity, with possible consequences for their future health. In addition, survey respondents reported neuropsychiatric disorders, including lack of concentration, memory deficits and difficulty processing information – factors that could affect children's academic performance and development. The mechanism by which these disturbances are generated is still unknown. Several hypotheses have been put forth, such as persistence of the virus in immunological sanctuaries, direct damage to organs, impaired coagulation with subsequent strokes and dysregulation of the immune system. We will probably understand the pathogenesis in the future, and we will be able to develop therapies capable of counteracting these disabling symptoms.

**Summary points**

- The presence of prolonged symptoms following acute COVID-19 infection (long COVID) is now known in the scientific community.
- It is still unclear whether long COVID can also affect children.
- The authors' study reported symptoms such as fatigue, headache, muscle and joint pain, lack of concentration and short-term memory problems.
- The authors' study provides further evidence of long COVID in children.
- Practitioners must not label the presence of prolonged symptoms after COVID-19 infection as mental health disorders, but rather suspect the presence of long COVID.
- Further studies are needed to improve the understanding and treatment of this disease.
