# Supplementary material for: Clinical characteristics, activity levels and mental health problems in children with long coronavirus disease: a survey of 510 children
Source: Future Microbiol. 2022 Apr 1;17(8):577–88. doi: 10.2217/fmb-2021-0285 (PMC9248023; doi:10.2217/fmb-2021-0285)
Supplement: Supplementary file 1 [file fmb-17-577-s1.docx]

**Supplementary materials**

| **Precondition** | **Freq** | **%** |
| --- | --- | --- |
| **Abdominal migraines** | 2 | 0.4% |
| **ADHD/ADS** | 28 | 5.5% |
| **Allergies** | 81 | 15.9% |
| **Anxiety** | 38 | 7.5% |
| **Asthma** | 74 | 14.5% |
| **Autism** | 29 | 5.7% |
| **Cerebral Palsy** | 2 | 0.4% |
| **Coeliac** | 8 | 1.6% |
| **Downs Syndrome** | 3 | 0.6% |
| **Dyspraxia** | 6 | 1.2% |
| **Eczema** | 63 | 12.4% |
| **EDS** | 6 | 1.2% |
| **Epilepsy** | 9 | 1.8% |
| **Epstein Bar** | 6 | 1.2% |
| **Gluten sensitivity** | 2 | 0.4% |
| **Hayfever** | 2 | 0.4% |
| **Headaches** | 36 | 7.1% |
| **Heart disease** | 2 | 0.4% |
| **Heart murmur** | 2 | 0.4% |
| **HPV Virus** | 2 | 0.4% |
| **HSV virus** | 2 | 0.4% |
| **Hyper-mobility** | 51 | 10% |
| **Hypertonia** | 5 | 1% |
| **IBS** | 4 | 0.8% |
| **Lyme Disease** | 3 | 0.6% |
| **OCD/Depression/Anxiety** | 36 | 7.1% |
| **Pandas** | 2 | 0.4% |
| **POT’s** | 6 | 1.2% |
| **Sensory Processing Disorder** | 4 | 0.8% |
| **Stomach pain** | 2 | 0.4% |
| **TICS/Tourettes** | 3 | 0.6% |
| **Urticaria** | 2 | 0.4% |
| **Other** | 48 | 9.4% |
| **None** | 223 | 43.7% |

**Table S1.** Pre-existing conditions before COVID-19 infection (multiple choices and free text allowed). Conditions found only in 1 Long-COVID child were grouped into Other

| **Symptom** | **All** | **Clinical Diagnosis** | **Positive Test** | **Unconfirmed but Suspected** |
| --- | --- | --- | --- | --- |
| **Cardio-respiratory** |  |  |  |  |
| Heart palpitations | 205 (40.2%) | 71 (45.5%) | 61 (42.1%) | 73 (34.9%) |
| Coughing | 151 (29.6%) | 52 (33.3%) | 43 (29.7%) | 56 (26.8%) |
| Throat clearing | 107 (21%) | 30 (19.2%) | 30 (20.7%) | 47 (22.5%) |
| **Dermatologic** |  |  |  |  |
| A rash | 267 (52.4%) | 83 (53.2%) | 73 (50.3%) | 111 (53.1%) |
| Red and cracked lips | 201 (39.4%) | 69 (44.2%) | 53 (36.6%) | 79 (37.8%) |
| Peeling skin on your hands and feet | 143 (28%) | 52 (33.3%) | 37 (25.5%) | 54 (25.8%) |
| Swollen hands and feet | 107 (21%) | 43 (27.6%) | 24 (16.6%) | 40 (19.1%) |
| Ulcers | 79 (15.5%) | 24 (15.4%) | 17 (11.7%) | 38 (18.2%) |
| **Gastrointestinal** |  |  |  |  |
| Tummy pain or cramps | 387 (75.9%) | 124 (79.5%) | 111 (76.6%) | 152 (72.7%) |
| Nausea | 233 (45.7%) | 80 (51.3%) | 67 (46.2%) | 86 (41.1%) |
| Diarrhea and vomiting | 216 (42.4%) | 78 (50%) | 67 (46.2%) | 71 (34%) |
| **HEENT (Head, ears, eyes, nose, throat)** |  |  |  |  |
| Red eyes | 206 (40.4%) | 74 (47.4%) | 55 (37.9%) | 77 (36.8%) |
| Sore throat | 230 (45.1%) | 80 (51.3%) | 60 (41.4%) | 90 (43.1%) |
| Swollen neck glands | 128 (25.1%) | 49 (31.4%) | 36 (24.8%) | 43 (20.6%) |
| **Musculoskeletal** |  |  |  |  |
| Muscle aches and pains | 349 (68.4%) | 117 (75%) | 102 (70.3%) | 130 (62.2%) |
| Muscle and joint pain | 309 (60.6%) | 103 (66%) | 92 (63.4%) | 114 (54.5%) |
| **Neurological** |  |  |  |  |
| Headache | 401 (78.6%) | 131 (84%) | 116 (80%) | 154 (73.7%) |
| Unexplained irritability | 262 (51.4%) | 90 (57.7%) | 70 (48.3%) | 102 (48.8%) |
| Dizzyness | 245 (48%) | 82 (52.6%) | 77 (53.1%) | 86 (41.1%) |
| Twitches | 55 (10.8%) | 17 (10.9%) | 16 (11%) | 22 (10.5%) |
| Word repetition | 52 (10.2%) | 20 (12.8%) | 16 (11%) | 16 (7.7%) |
| Tics | 47 (9.2%) | 18 (11.5%) | 8 (5.5%) | 21 (10%) |
| Stuttering | 40 (7.8%) | 11 (7.1%) | 10 (6.9%) | 19 (9.1%) |
| Swearing | 26 (5.1%) | 11 (7.1%) | 4 (2.8%) | 11 (5.3%) |
| Growling | 24 (4.7%) | 9 (5.8%) | 6 (4.1%) | 9 (4.3%) |
| **General** |  |  |  |  |
| Tiredness and weakness | 444 (87.1%) | 139 (89.1%) | 131 (90.3%) | 174 (83.3%) |
| Fatigue | 410 (80.4%) | 139 (89.1%) | 116 (80%) | 155 (74.2%) |
| Post-exertional malaise | 274 (53.7%) | 110 (70.5%) | 71 (49%) | 93 (44.5%) |
| Fever | 151 (29.6%) | 63 (40.4%) | 45 (31%) | 43 (20.6%) |
| Flu-like symptoms | 121 (23.7%) | 49 (31.4%) | 37 (25.5%) | 35 (16.7%) |
| **Other** |  |  |  |  |
| Sepsis | 7 (1.4%) | 2 (1.3%) | 1 (0.7%) | 4 (1.9%) |
| Appendicitis | 7 (1.4%) | 2 (1.3%) | 1 (0.7%) | 4 (1.9%) |
| Peritonitis | 1 (0.2%) | 1 (0.6%) | 0 (0%) | 0 (0%) |

**Table S2.** Symptoms present since COVID-19 infection (multiple choices allowed), by confirmation status of COVID-19 infection.

| **Changed since COVID** | **All** | **Clinical Diagnosis** | **Positive Test** | **Unconfirmed but Suspected** | **Had Pre-conditions** | **No Pre-conditions** |
| --- | --- | --- | --- | --- | --- | --- |
| **Appetite** | 253 (49.6%) | 93 (59.6%) | 72 (49.7%) | 88 (42.1%) | 146 (50.9%) | 107 (48%) |
| **Behaviour** | 166 (32.5%) | 46 (29.5%) | 40 (27.6%) | 80 (38.3%) | 91 (31.7%) | 75 (33.6%) |
| **Cognitive function** | 197 (38.6%) | 75 (48.1%) | 56 (38.6%) | 66 (31.6%) | 126 (43.9%) | 71 (31.8%) |
| **Energy levels** | 425 (83.3%) | 137 (87.8%) | 127 (87.6%) | 161 (77%) | 241 (84%) | 184 (82.5%) |
| **Eye sight** | 110 (21.6%) | 39 (25%) | 25 (17.2%) | 46 (22%) | 63 (22%) | 47 (21.1%) |
| **Hearing** | 36 (7.1%) | 15 (9.6%) | 4 (2.8%) | 17 (8.1%) | 18 (6.3%) | 18 (8.1%) |
| **Mental health** | 188 (36.9%) | 56 (35.9%) | 60 (41.4%) | 72 (34.4%) | 115 (40.1%) | 73 (32.7%) |
| **Mobility** | 187 (36.7%) | 70 (44.9%) | 50 (34.5%) | 67 (32.1%) | 113 (39.4%) | 74 (33.2%) |
| **Mood** | 300 (58.8%) | 89 (57.1%) | 87 (60%) | 124 (59.3%) | 173 (60.3%) | 127 (57%) |
| **Skin** | 189 (37.1%) | 64 (41%) | 47 (32.4%) | 78 (37.3%) | 109 (38%) | 80 (35.9%) |
| **Sleep** | 287 (56.3%) | 95 (60.9%) | 91 (62.8%) | 101 (48.3%) | 164 (57.1%) | 123 (55.2%) |
| **Toilet habits** | 124 (24.3%) | 45 (28.8%) | 32 (22.1%) | 47 (22.5%) | 67 (23.3%) | 57 (25.6%) |

**Table S3.** Changes reported since COVID infection, by confirmation status of COVID-19 infection, and by the pre-existence of comorbidity conditions

| **Number of changes since COVID-19** | **All** | **Clinical Diagnosis** | **Positive Test** | **Unconfirmed but suspected** | **Had Pre-Conditions** | **No Pre-Conditions** |
| --- | --- | --- | --- | --- | --- | --- |
| **1** | 63 (12.4%) | 16 (10.3%) | 15 (10.3%) | 32 (15.3%) | 31 (10.8%) | 32 (14.3%) |
| **2** | 59 (11.6%) | 15 (9.6%) | 17 (11.7%) | 27 (12.9%) | 31 (10.8%) | 28 (12.6%) |
| **3** | 63 (12.4%) | 20 (12.8%) | 17 (11.7%) | 26 (12.4%) | 38 (13.2%) | 25 (11.2%) |
| **4 or more** | 325 (63.7%) | 105 (67.3%) | 96 (66.2%) | 124 (59.3%) | 187 (65.2%) | 138 (61.9%) |

**Table S4.** Number of changes since COVID-19 infection, by confirmation status of infection, and by the pre-existence of comorbidity conditions

| **Activity before infection** | **Activity first 6 weeks** | | | |
| --- | --- | --- | --- | --- |
|  | **No** | **Unsure** | **Yes** | **Total** |
| **Daily Sports/Dance** | 44 | 4 | 96 | 144 |
| **Weekly Sports/Dance** | 77 | 10 | 92 | 179 |
| **Weekly Moderate Activity** | 76 | 13 | 71 | 160 |
| **Very Occasional Activity** | 13 | 4 | 1 | 18 |
| **Rare Activity** | 7 | 0 | 2 | 9 |

**Table S5.** Activity level before infection by Participation in Any level of activity in the first 6 weeks of COVID-19 infection

| **Current Activity** | **Activity before infection** | | | | |  |
| --- | --- | --- | --- | --- | --- | --- |
|  | **Daily Sports/Dance** | **Weekly Sports/Dance** | **Weekly Moderate** | **Very Occasional** | **Rare Activity** | **Total** |
| **Returned to previous** | 17 | 15 | 18 | 1 | 0 | 51 |
| **Level varies by day** | 38 | 59 | 58 | 6 | 4 | 165 |
| **Occasional, same symptoms** | 10 | 9 | 9 | 4 | 0 | 32 |
| **Occasional, worst symptoms** | 44 | 59 | 46 | 2 | 3 | 154 |
| **Unable to enjoy** | 35 | 37 | 29 | 5 | 2 | 108 |

**Table S6.** Child’s current activity level by Activity level before infection

| **Mental Health / Cognitive Issues** | **All** | **Clinical Diagnosis** | **Positive Test** | **Unconfirmed but Suspected** | **Had Pre-conditions** | **No Pre-conditions** |
| --- | --- | --- | --- | --- | --- | --- |
| **Difficulty in doing everyday tasks** | 204 (40%) | 79 (50.6%) | 61 (42.1%) | 64 (30.6%) | 122 (42.5%) | 82 (36.8%) |
| **Difficulty processing information** | 167 (32.7%) | 62 (39.7%) | 49 (33.8%) | 56 (26.8%) | 110 (38.3%) | 57 (25.6%) |
| **Difficulty remembering information** | 234 (45.9%) | 88 (56.4%) | 66 (45.5%) | 80 (38.3%) | 140 (48.8%) | 94 (42.2%) |
| **Difficulty understanding instruction** | 147 (28.8%) | 57 (36.5%) | 35 (24.1%) | 55 (26.3%) | 86 (30%) | 61 (27.4%) |
| **Finds it difficult to make a decision** | 139 (27.3%) | 46 (29.5%) | 38 (26.2%) | 55 (26.3%) | 81 (28.2%) | 58 (26%) |
| **Finds it hard to work out what order to do things in** | 89 (17.5%) | 30 (19.2%) | 28 (19.3%) | 31 (14.8%) | 55 (19.2%) | 34 (15.2%) |
| **Forgets what they are saying** | 146 (28.6%) | 54 (34.6%) | 46 (31.7%) | 46 (22%) | 91 (31.7%) | 55 (24.7%) |
| **Hesitating before speaking or moving** | 102 (20%) | 34 (21.8%) | 32 (22.1%) | 36 (17.2%) | 58 (20.2%) | 44 (19.7%) |
| **Lack of concentration** | 309 (60.6%) | 108 (69.2%) | 89 (61.4%) | 112 (53.6%) | 180 (62.7%) | 129 (57.8%) |
| **Often has a vacant look** | 107 (21%) | 31 (19.9%) | 44 (30.3%) | 32 (15.3%) | 67 (23.3%) | 40 (17.9%) |
| **Short term memory issues** | 167 (32.7%) | 66 (42.3%) | 47 (32.4%) | 54 (25.8%) | 108 (37.6%) | 59 (26.5%) |
| **Struggles to choose appropriate clothes** | 54 (10.6%) | 16 (10.3%) | 16 (11%) | 22 (10.5%) | 31 (10.8%) | 23 (10.3%) |
| **Struggles to find the right words** | 162 (31.8%) | 65 (41.7%) | 41 (28.3%) | 56 (26.8%) | 95 (33.1%) | 67 (30%) |
| **Stuttering** | 40 (7.8%) | 9 (5.8%) | 14 (9.7%) | 17 (8.1%) | 19 (6.6%) | 21 (9.4%) |
| **Uses the wrong words for people or objects** | 73 (14.3%) | 26 (16.7%) | 21 (14.5%) | 26 (12.4%) | 45 (15.7%) | 28 (12.6%) |
| **None of the above** | 111 (21.8%) | 24 (15.4%) | 29 (20%) | 58 (27.8%) | 57 (19.9%) | 54 (24.2%) |
| **Other** | 53 (10.4%) | 18 (11.5%) | 11 (7.6%) | 24 (11.5%) | 33 (11.5%) | 20 (9%) |

**Table S7.** Mental health / cognitive issues since COVID-19 infection (multiple issues per child are usual), by confirmation status of infection, and by the pre-existence of comorbidity conditions

| **Number of issues since COVID-19** | **All** | **Clinical Diagnosis** | **Positive Test** | **Unconfirmed but suspected** | **Had Pre-Conditions** | **No Pre-Conditions** |
| --- | --- | --- | --- | --- | --- | --- |
| **0** | 132 (25.9%) | 28 (17.9%) | 32 (22.1%) | 72 (34.4%) | 68 (23.7%) | 64 (28.7%) |
| **1** | 54 (10.6%) | 17 (10.9%) | 18 (12.4%) | 19 (9.1%) | 29 (10.1%) | 25 (11.2%) |
| **2** | 45 (8.8%) | 9 (5.8%) | 12 (8.3%) | 24 (11.5%) | 18 (6.3%) | 27 (12.1%) |
| **3 or more** | 279 (54.7%) | 102 (65.4%) | 83 (57.2%) | 94 (45%) | 172 (59.9%) | 107 (48%) |

**Table S8.** Number of mental health / cognitive issues since COVID-19 infection (from those in Table 5, excluding “None of the above” and “Other”), by confirmation status of infection, and by the pre-existence of comorbidity conditions


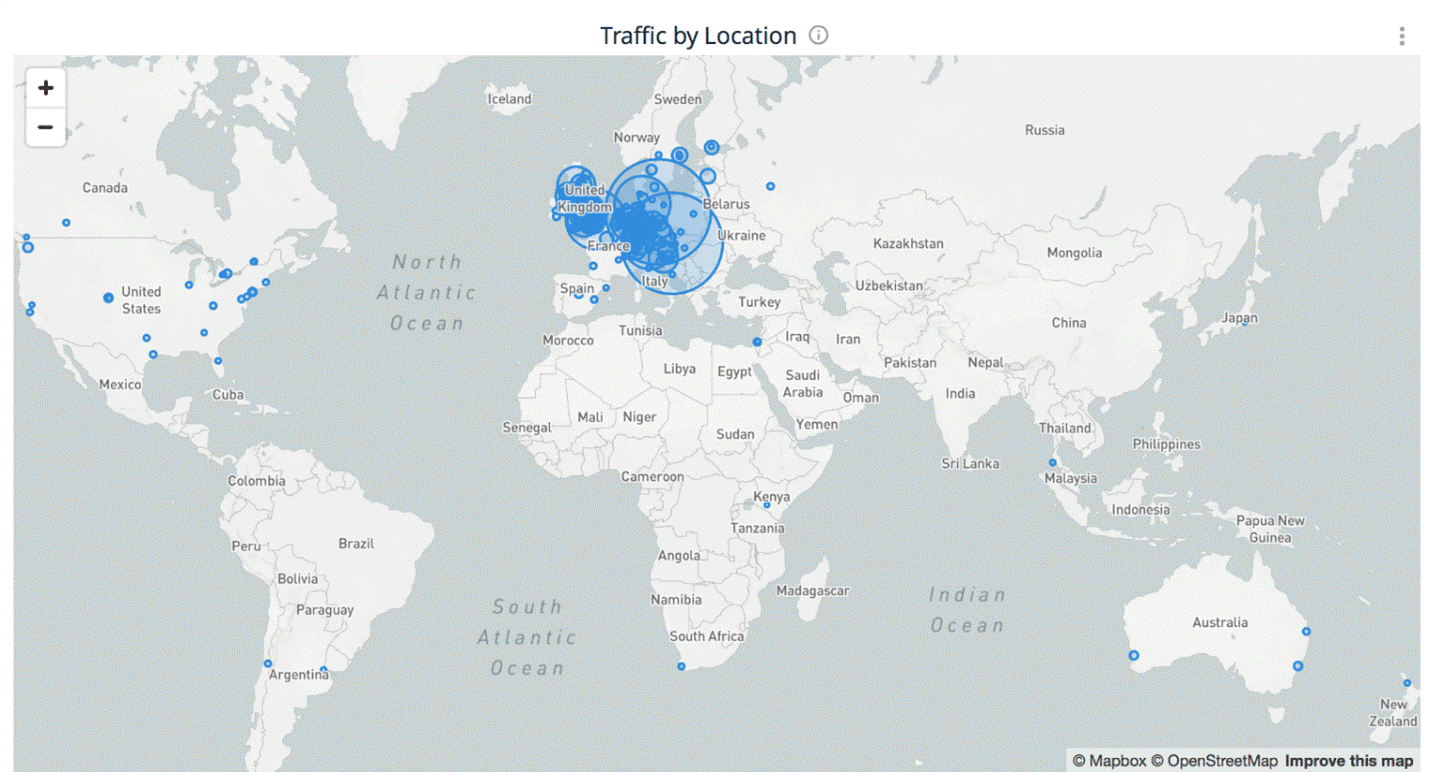


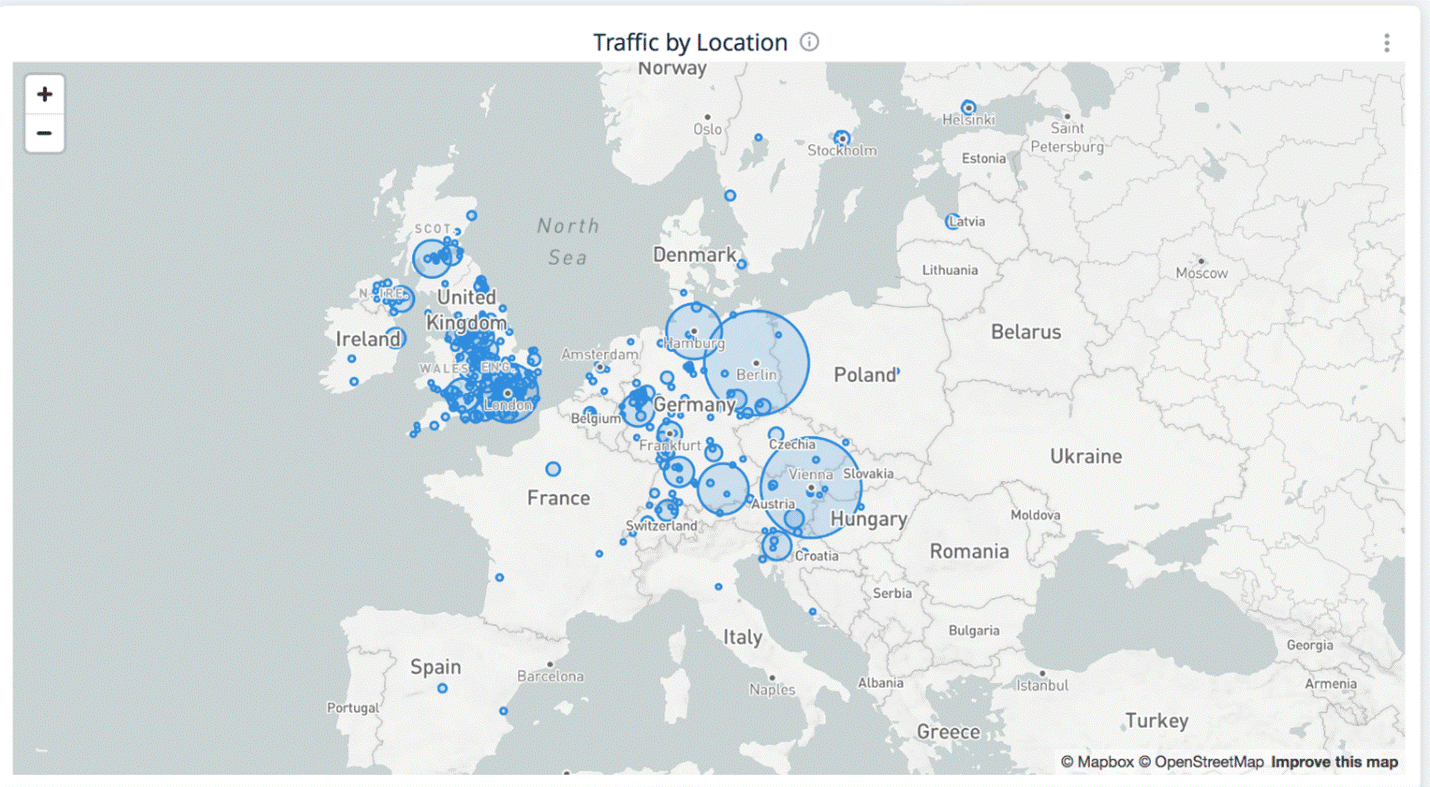


Supplemental Figure S1. Social media traffic of the Long Covid Kids UK platform.
